# Severe Loneliness Among Community-Dwelling Older Adults at Risk of Falls in Andalusia: Epidemiological Determinants and Clinical Correlates of Pain and Sleep Quality

**DOI:** 10.3390/healthcare14121753

**Published:** 2026-06-18

**Authors:** Gregorio Jesús Alcalá-Albert, María de la Soledad Guerrero-Alonso, Azahara Leonor Miranda-Gálvez, Gloria Marlén Aldana-de Becerra, José Hernández-Ascanio, Eduardo José Sánchez-Uzcategui

**Affiliations:** 1 Department of Health and Biomedical Sciences, Loyola University of Andalusia, 14004 Córdoba, Spain; gjalcala@uloyola.es (G.J.A.-A.); ejsanchez@uloyola.es (E.J.S.-U.); 2 Department of Psychology, Loyola University of Andalusia, 14004 Córdoba, Spain; mguerrero@uloyola.es (M.d.l.S.G.-A.); almiranda@uloyola.es (A.L.M.-G.); 3 Psychohomophilic Research Group, Ministry of National Education of Colombia, Bogotá 111321, Colombia; galdana415@yahoo.com

**Keywords:** loneliness, social determinants of health, older adults, sleep quality, pain, primary health care, Andalusia, epidemiology

## Abstract

**Highlights:**

**What are the main findings?**

- Severe loneliness was identified in 21.5% of community-dwelling older adults at risk of falls in Andalusia after correcting the scoring direction and categorisation of the UCLA Loneliness Scale.
- Severe loneliness was associated with a more vulnerable sociodemographic and clinical profile, including female sex, living alone, lower economic level, poorer family relationships, poorer sleep quality, severe pain, history of depression, lower physical exercise and fewer outings from home.

**What are the implications of the main findings?**

- Loneliness screening in primary care should be considered particularly relevant among clinically vulnerable older adults, especially those presenting frailty-related indicators, pain, sleep disturbances or reduced social participation.
- The observed associations support the need for multidimensional assessment strategies in community and primary care settings, although longitudinal and interventional studies are required before causal or therapeutic effects can be inferred.

**Abstract:**

**Background/Objectives**: Loneliness is a relevant social determinant of health in older age and has been associated with adverse physical, psychological and social outcomes. This study aimed to estimate the prevalence of severe loneliness and to identify its epidemiological and clinical correlates among community-dwelling older adults at risk of falls in Andalusia, Spain. **Methods**: A cross-sectional analytical study was conducted with 237 adults aged 65 years and older living in private households. Loneliness was assessed using the 10-item version of the UCLA Loneliness Scale, which showed excellent internal consistency in this sample (Cronbach’s alpha = 0.900). After reviewing the scoring direction of the instrument, severe loneliness was operationally defined using the corrected UCLA total score, with higher scores indicating greater loneliness. Sociodemographic and clinical variables included age, sex, living arrangements, economic level, family relationships, pain, sleep quality, depression history, physical exercise, outings from home, polymedication and analgesic use. Bivariate analyses were performed to compare participants with and without severe loneliness, and logistic regression was used to examine independent correlates of severe loneliness. **Results**: Severe loneliness was identified in 51 participants, corresponding to 21.5% of the analytical sample. The corrected UCLA-10 loneliness burden score ranged from 0 to 30, with higher scores indicating greater loneliness. Participants with severe loneliness were more likely to report poorer sleep quality and severe pain. In the adjusted logistic regression model including age, sex, sleep quality and severe pain, better sleep quality was associated with lower odds of severe loneliness (OR = 0.46, 95% CI: 0.30–0.72, *p* = 0.001), while severe pain was associated with higher odds of severe loneliness (OR = 3.15, 95% CI: 1.56–6.35, *p* = 0.001). Age (OR = 1.03, 95% CI: 0.99–1.07, *p* = 0.184) and female sex (OR = 2.00, 95% CI: 0.83–4.81, *p* = 0.124) were not statistically significant in the fully adjusted model. **Conclusions**: Severe loneliness affected a clinically relevant proportion of community-dwelling older adults at risk of falls in Andalusia. The findings should be interpreted as cross-sectional associations and not as evidence of causal pathways. Sleep quality and severe pain emerged as the main independent clinical correlates of severe loneliness in the adjusted model, supporting the relevance of multidimensional assessment in frail older adults.

## 1. Introduction

The demographic landscape of the 21st century is defined by an unprecedented global phenomenon: the rapid ageing of the human population. In Spain, and particularly within the Autonomous Community of Andalusia, this transition has reached a critical juncture. According to recent demographic projections, the proportion of individuals aged 65 and over is expected to increase significantly over the next few decades, placing immense pressure on both social structures and healthcare systems [1,2,3,4]. Within this context, loneliness among community-dwelling older adults has emerged not merely as a psychosocial distress but as a profound social determinant of health (SDH) with epidemiological consequences comparable to well-established risk factors such as smoking or obesity [5,6,7].

Loneliness is defined as the subjective, distressing experience resulting from a perceived discrepancy between an individual’s desired and actual social relationships [8,9,10]. It is imperative to distinguish this from social isolation, which refers to an objective lack of social contacts or roles [10]. While related, these constructs operate through different pathways; an older adult may live in a densely populated household yet experience profound loneliness, or, conversely, live alone while remaining socially satisfied [11]. However, in the epidemiological study of ageing, the most severe forms of loneliness—characterised by chronic feelings of exclusion and a lack of relational reciprocity—have been identified as primary drivers of morbidity [12,13].

The impact of severe loneliness on physical health is mediated through complex physiological and behavioural mechanisms. Chronic loneliness has been associated with dysregulation of the hypothalamic–pituitary–adrenal (HPA) axis, increased systemic inflammation (as evidenced by elevated C-reactive protein levels), and impaired immune function [12,14]. Furthermore, it acts as a significant predictor of cardiovascular disease, cognitive decline, and all-cause mortality among the elderly [7,13,14,15,16,17]. In Andalusia, a region with deep-seated traditions of family-based care, the erosion of traditional support networks due to urbanisation and changing household compositions has created a “vulnerability gap”, where older adults are increasingly exposed to social disconnection [1,3].

Recent literature has highlighted that loneliness does not affect the elderly population uniformly. Rather, it is patterned by structural inequalities, most notably gender [18]. Women often report higher levels of loneliness than men, a disparity frequently attributed to longer life expectancy, a higher prevalence of widowhood, and life trajectories marked by economic disadvantages and caregiving burdens [18,19]. However, some researchers argue that men may under-report loneliness due to social stigma, suggesting that the epidemiological burden may be more complex than self-reported data initially indicate [18]. In the Andalusian context, where the “feminisation of ageing” is pronounced, understanding the gendered nature of loneliness is essential for designing targeted clinical interventions [1].

A significant gap in current research involves the intersection between severe loneliness and modifiable clinical factors, specifically chronic pain and sleep quality. While loneliness is often studied as a predictor of mental health outcomes like depression, its relationship with somatic symptoms is equally critical [11]. Chronic pain, particularly musculoskeletal in origin, is highly prevalent among older adults [20,21,22,23,24,25,26,27]. Pain not only restricts physical mobility and community participation—thereby increasing isolation—but also intensifies the subjective experience of being alone [25,26,27]. Emerging evidence suggests a bidirectional relationship: loneliness may lower pain thresholds through emotional dysregulation, while persistent pain serves as a barrier to maintaining the social bonds that protect against loneliness [25,26,27].

Similarly, sleep quality has been identified as a robust clinical correlate of social well-being. Older adults frequently experience age-related sleep disturbances, but severe loneliness appears to exacerbate these issues through the mechanism of “social hypervigilance” [21,22]. Subjective social disconnection creates a state of heightened arousal, leading to fragmented sleep and poor sleep hygiene, which in turn impairs the cognitive and emotional resources necessary for social engagement the following day [22,23]. From a clinical perspective, identifying these modifiable factors—pain and sleep—offers a pathway for healthcare providers to intervene in social vulnerability through physiological management [21,24].

Despite the growing body of international evidence, there is a scarcity of recent, large-scale epidemiological data specifically focused on the Andalusian population during the post-pandemic era. The year 2026 presents a unique opportunity to evaluate these determinants, as the long-term effects of social shifts and the digitalisation of communication have fundamentally altered the relational environment for the elderly [1,20]. Previous studies in the region have often relied on small convenience samples or lacked multivariable adjustments for clinical comorbidities [1,3]. Consequently, there is an urgent need to establish the prevalence of severe loneliness in this population and to identify which clinical and epidemiological markers most accurately predict this state.

This study is grounded in the framework of Social Epidemiology, viewing loneliness not as an individual failure but as a product of the interaction between the individual and their sociosanitary environment [6,10,15]. In Andalusia, the primary care system serves as the first line of defence against the complications of ageing [1,3]. However, screening for loneliness remains inconsistent, often overshadowed by the management of acute or chronic physical ailments [28,29,30,31,32,33]. By establishing the link between severe loneliness and modifiable clinical indicators like sleep quality and pain, this research aims to provide primary care professionals, particularly community nurses and general practitioners, with empirical evidence to support a more integrated approach to geriatric health [21,25,26,27,33].

The present study, conducted during the first quarter of 2026, focuses on community-dwelling older adults in Andalusia who are at risk of falls—a group representing a high level of physical, functional and social vulnerability [4,28,29]. The choice of this specific subpopulation is justified by the established link between mobility limitations, fall-related vulnerability, reduced participation outside the home and social disconnection [28,29,30,31,32,33,34,35]. We hypothesised that severe loneliness would be associated with a broader profile of sociodemographic, clinical and functional vulnerability, particularly sex, age, sleep quality and pain.

The primary objective of this research is twofold: first, to estimate the prevalence of severe loneliness among community-dwelling older adults at risk of falls in Andalusia using a validated psychometric instrument; second, to identify the epidemiological and clinical correlates—specifically age, sex, pain and sleep quality—associated with severe loneliness. By doing so, this study seeks to contribute to the international literature on the social determinants of health and to provide evidence for multidimensional assessment strategies in primary care and community health settings.

## 2. Materials and Methods

### 2.1. Study Design and Data Source

This study employed a cross-sectional, analytical, observational design to examine the epidemiological determinants and clinical correlates of severe loneliness among community-dwelling older adults. The research was conducted during the first quarter of 2026 (January to March) across the Autonomous Community of Andalusia, Spain. The study was designed and reported in accordance with the Strengthening the Reporting of Observational Studies in Epidemiology (STROBE) guidelines to ensure methodological transparency and scientific rigour [36].

The investigation was situated within the theoretical framework of Social Epidemiology, which conceptualises loneliness not as an isolated psychological state but as a socially patterned determinant of health [6,10,15]. This approach allows for the analysis of individual clinical symptoms, such as pain and sleep disturbances, as being embedded within broader sociodemographic and structural contexts. By employing a multivariable analytical strategy, the study aimed to isolate the independent contribution of modifiable clinical factors while adjusting for traditional epidemiological variables such as age and sex.

### 2.2. Participants

The study was conducted within the primary care network of the Andalusian Public Health System. The target population consisted of non-institutionalised adults aged 65 years and older residing in private households in Andalusia and presenting fall-related vulnerability. Therefore, the study population should not be interpreted as representative of all community-dwelling older adults in Andalusia, but rather as a clinically selected subgroup of older adults at risk of falls.

Participants were recruited through primary care and community-based contacts. The recruitment strategy was designed to identify older adults living in the community who met the predefined clinical inclusion criteria, particularly fall-related vulnerability. Although a probabilistic regional sampling design was considered during the planning phase, the final recruitment process was constrained by fieldwork feasibility and access to eligible participants. Consequently, the final analytical sample should be interpreted as a clinically selected sample of community-dwelling older adults at risk of falls rather than as a fully representative probabilistic sample of the Andalusian older adult population.

The final analytical sample consisted of 237 older adults. To ensure the homogeneity of the clinical profile, the following eligibility criteria were applied:

- Inclusion criteria: (a) age 65 years or older; (b) residence in a private household within Andalusia; and (c) fall-related vulnerability, defined as a score of 1 or above on the fall-risk screening questionnaire.
- Exclusion criteria: (a) moderate to severe cognitive impairment that would preclude the accurate completion of psychometric scales; (b) institutionalisation in nursing homes or long-term care facilities; and (c) refusal to provide informed consent.

### 2.3. Variables

Data were collected using a structured data collection form (DCF) that included self-reported sociodemographic and clinical variables.

Loneliness (Dependent Variable): Loneliness was assessed using the 10-item version of the UCLA Loneliness Scale [9,36,37]. In the database, item responses had been recoded so that higher item values reflected lower loneliness burden. To obtain a score interpretable in the standard direction, where higher values indicate greater loneliness, a corrected UCLA-10 loneliness burden score was calculated as follows: corrected UCLA-10 score = 40 − total recoded loneliness score. This transformation produced a total score ranging from 0 to 30, corresponding to 10 items scored from 0 to 3. The internal consistency of the corrected scale was excellent (Cronbach’s alpha = 0.900). Severe loneliness was operationally defined as a corrected UCLA-10 score of 21 or higher, moderate loneliness as a score between 10 and 20, and mild or minimal loneliness as a score between 0 and 9. For the logistic regression analysis, severe loneliness was dichotomised as severe loneliness versus non-severe loneliness. This classification should be interpreted as an operational analytical category rather than as a formal clinical diagnosis.

Pain: Pain intensity was measured using the Visual Analogue Scale (VAS), ranging from 0, indicating no pain, to 10, indicating the worst imaginable pain [38]. Severe pain was operationalised according to the predefined clinical threshold used in the database and was included in the multivariable model because of its theoretical relevance and strong bivariate association with severe loneliness.

Sleep quality: Sleep quality was assessed using a self-reported Likert-type item. Higher scores indicated better perceived sleep quality. This variable should therefore be interpreted as a subjective measure of perceived sleep quality rather than as a diagnostic assessment of sleep disorder.

Risk of falls: Risk of falls was assessed using a standardised screening questionnaire. A score of 1 or above was required for inclusion in the study, as the target population consisted of community-dwelling older adults with fall-related vulnerability [4,39].

Other independent variables: Additional sociodemographic, clinical and functional variables included age, sex, mood, socioeconomic status, living arrangements, family relationships, frequency of physical exercise, outings from home, depression history, multimorbidity, polymedication and analgesic use. These variables were collected using the structured data collection form and were analysed as indicators of the participants’ broader clinical, functional and social vulnerability.

### 2.4. Statistical Analysis

Data analysis was performed using Python (version 3.10) with the SciPy and Statsmodels libraries, as well as G-Stat (v.2). The analysis followed a pre-defined three-phase protocol:

Descriptive and Reliability Analysis: Frequencies and percentages were calculated for categorical variables, while means and standard deviations were used for continuous data. The internal consistency of the UCLA scale was verified using Cronbach’s alpha.

Bivariate Comparisons: To explore the relationship between severe loneliness and predictors, group comparisons were conducted using the t-test for normally distributed continuous variables and the Mann–Whitney U test for non-parametric data. Categorical associations were tested using Fisher’s exact test or Chi-square test.

Multivariable Modelling: A multivariable logistic regression model was estimated with severe loneliness as the dependent variable. Severe loneliness was defined as a corrected UCLA-10 loneliness burden score of 21 or higher. Age, sex, sleep quality and severe pain were included as independent variables because of their theoretical relevance and their role in the study objectives. Adjusted odds ratios (OR), 95% confidence intervals (CI) and *p*-values were reported. Collinearity diagnostics did not indicate problematic multicollinearity among the predictors included in the model. Given the cross-sectional design, regression coefficients were interpreted as measures of statistical association and not as evidence of causal effects or temporal relationships.

### 2.5. Use of Generative Artificial Intelligence

During the preparation of this manuscript, the authors used ChatGPT (OpenAI, GPT-5.5 Thinking) to assist with manuscript drafting, structuring according to the MDPI template, statistical reporting, and language editing. The authors reviewed and edited all outputs and take full responsibility for the content of this publication. The tool was not used to generate primary data.

## 3. Results

### 3.1. Sample Characteristics and Epidemiological Profile

The final analytic sample comprised 237 community-dwelling older adults. The mean age of the participants was 79.35 years (SD = 8.36; range: 65–98). The sample exhibited a significant feminisation of ageing, with 68.8% (*n* = 163) being women. Regarding clinical comorbidities, the population showed high levels of frailty; notably, 100% of participants presented with a significant gait disorder and were at risk of falls. Furthermore, 79.7% were polymedicated, and 77.6% suffered from chronic musculoskeletal conditions.

### 3.2. Prevalence and Symptom Burden of Severe Loneliness

After correcting the scoring direction of the UCLA-10 Loneliness Scale, the corrected loneliness burden score ranged from 0 to 30, with higher scores indicating greater loneliness. The mean corrected UCLA-10 score was 15.62 (SD = 6.74). Severe loneliness, defined as a corrected UCLA-10 score of 21 or higher, a value equal to or greater than 51 participants was identified, which corresponds to 21.5% of the analytical sample, as can be seen in Table 1.

Moderate loneliness was observed in 144 participants (60.8%), while 42 participants (17.7%) were classified as having mild or minimal loneliness.

This corrected estimate indicates that severe loneliness was present in a clinically relevant minority of community-dwelling older adults at risk of falls in Andalusia. Therefore, the prevalence should not be interpreted as affecting the majority of the sample or as representative of all community-dwelling older adults in the region. Instead, it describes the burden of severe loneliness within a clinically selected subgroup of older adults with fall-related vulnerability.

The item-level analysis, which can be seen in Table 2, showed that the highest loneliness burden corresponded to tolerance of solitude (mean = 2.10, SD = 0.91), waiting for calls or letters (mean = 1.94, SD = 0.89), inability to communicate (mean = 1.88, SD = 0.96), desire for company (mean = 1.86, SD = 0.94), and unhappiness doing things alone (mean = 1.77, SD = 0.89).

These findings suggest that loneliness in this clinically vulnerable population was closely related to perceived deficits in relational reciprocity, availability of contact and meaningful communication.

### 3.3. Bivariate Clinical Correlates

In the bivariate analysis, participants with severe loneliness showed a more vulnerable sociodemographic and clinical profile than those without severe loneliness. Severe loneliness was more frequent among women and among participants living alone. It was also associated with lower economic level, poorer family relationships, poorer sleep quality, severe pain, history of depression, lower physical exercise and fewer outings from home.

These findings suggest that severe loneliness in this population was not solely related to objective living arrangements, but also to a broader constellation of social, clinical and functional vulnerabilities.

### 3.4. Multivariable Correlates of Severe Loneliness

A multivariable logistic regression model was estimated to identify factors independently associated with severe loneliness. The results of this model are shown in Table 3. Severe loneliness was defined as a corrected loneliness burden score of 21 or higher on the UCLA-10 scale. The model included age, sex, sleep quality, and severe pain.

In the adjusted model, better perceived sleep quality was associated with lower odds of severe loneliness (OR = 0.46, 95% CI: 0.30–0.72, *p* = 0.001). Severe pain was associated with higher odds of severe loneliness (OR = 3.15, 95% CI: 1.56–6.35, *p* = 0.001). Age was not statistically significant in the fully adjusted model (OR = 1.03, 95% CI: 0.99–1.07, *p* = 0.184), and female sex was also not statistically significant after adjustment for sleep quality and severe pain (OR = 2.00, 95% CI: 0.83–4.81, *p* = 0.124).

These results indicate that sleep quality and severe pain were the main independent clinical correlates of severe loneliness in this sample of community-dwelling older adults at risk of falls. However, because of the cross-sectional design, these associations should not be interpreted as causal relationships.

## 4. Discussion

The present study examined the prevalence and clinical correlates of severe loneliness among community-dwelling older adults at risk of falls in Andalusia. Using a corrected UCLA-10 loneliness burden score ranging from 0 to 30, severe loneliness was identified in 21.5% of the analytical sample. This estimate indicates that severe loneliness represents a clinically relevant condition in this vulnerable subgroup, although it should not be interpreted as representative of the broader population of community-dwelling older adults in Andalusia. Rather, the prevalence should be understood in relation to the specific clinical profile of the participants, all of whom presented fall-related vulnerability.

The corrected prevalence is consistent with previous evidence showing that loneliness in later life is not randomly distributed, but is patterned by mental health, socioeconomic position, living arrangements, perceived social support, functional limitations and health-related vulnerability [10,11,15,30,31]. In this regard, the present findings should be interpreted within a social epidemiological framework, in which loneliness is understood not only as an individual emotional experience but also as a health-relevant condition embedded in structural, relational and clinical contexts [6,10,15].

A central contribution of this study is the explicit focus on older adults at risk of falls. Participants showed a high burden of frailty-related indicators, including gait disorder, polymedication and chronic musculoskeletal conditions. Therefore, the study population represents a clinically selected subgroup rather than a general community sample. This distinction is important because fall-related vulnerability may restrict mobility, reduce confidence in leaving the home, limit social participation and increase perceived social disconnection. Previous evidence has shown that falls and fall-related experiences may be associated with subsequent social isolation and loneliness in older adults [28,29]. Accordingly, the present findings support the need to consider loneliness within broader geriatric vulnerability profiles that include mobility limitation, pain, fear of falling and reduced participation outside the home.

The bivariate profile showed that severe loneliness was more frequent among women, participants living alone, those with lower economic level, poorer family relationships, history of depression, reduced physical exercise and fewer outings from home. These findings are consistent with previous studies showing that loneliness in older age is associated with socioeconomic disadvantage, depressive symptoms, lower quality of life and reduced social participation [11,19,30,31,33]. However, in the adjusted model, age and female sex were no longer statistically significant after accounting for sleep quality and severe pain. This suggests that, in this fall-risk subgroup, demographic markers may be less relevant than modifiable clinical and functional vulnerability when explaining the adjusted profile of severe loneliness.

The distinction between loneliness and social isolation remains essential for interpreting these findings. Living alone and reduced outings may indicate objective constraints on social participation, but loneliness refers to the subjective discrepancy between desired and actual social relationships [8,9,10]. This distinction is clinically important because older adults may experience severe loneliness even when they are not objectively isolated, while others may live alone without reporting severe loneliness. The item-level findings reinforce this interpretation: the highest burden was related to tolerance of solitude, waiting for calls or letters, inability to communicate, desire for company and unhappiness doing things alone. These dimensions suggest that severe loneliness in this sample was closely linked to perceived relational reciprocity, availability of contact and meaningful communication, rather than merely to household composition.

In the fully adjusted model, sleep quality and severe pain emerged as the main independent clinical correlates of severe loneliness. Better perceived sleep quality was associated with lower odds of severe loneliness, whereas severe pain was associated with higher odds. This pattern suggests that, among older adults at risk of falls, severe loneliness may be closely connected with modifiable clinical and functional conditions. Importantly, these findings should be interpreted as cross-sectional associations rather than causal effects.

The association between sleep quality and severe loneliness is consistent with previous evidence linking loneliness and social isolation with sleep disturbances in older adults [21,22,24]. Mechanistically, loneliness may contribute to heightened vigilance, fragmented sleep and poorer restorative rest, while poor sleep may reduce emotional regulation, energy and motivation for social engagement [12,21,22,23]. However, the present study cannot establish whether sleep problems preceded loneliness, resulted from loneliness or formed part of a reciprocal process. Sleep quality should therefore be interpreted as a clinically relevant correlate and potential marker of social and emotional vulnerability, not as evidence of a causal protective factor.

The adjusted association between severe pain and severe loneliness reinforces the importance of considering somatic and social vulnerability together. Chronic pain may restrict mobility, reduce participation outside the home, increase dependence on others and limit opportunities for meaningful social interaction. Conversely, loneliness may intensify the subjective experience of pain through psychological distress, reduced coping resources and altered affective processing [25,26,27]. The persistence of severe pain in the adjusted model supports its inclusion as a key clinical correlate of severe loneliness among older adults at risk of falls. Nevertheless, because the study is cross-sectional, the directionality of this association cannot be determined.

These results have implications for primary care and community health services. The findings do not demonstrate that improving sleep quality, pain management or mobility would necessarily reduce loneliness. However, sleep complaints, severe pain, reduced outings and fall-related vulnerability may function as warning signs for identifying older adults who require a more detailed assessment of loneliness and social vulnerability. This interpretation is aligned with public health recommendations recognising loneliness and social isolation among older people as relevant health and policy concerns [5,6,17]. It is also consistent with evidence indicating that interventions to reduce loneliness and social isolation in older adults are heterogeneous, usually modest in effect size, and dependent on target population, design, context and intervention components [34,35].

Overall, the present study supports the integration of loneliness screening into multidimensional geriatric and primary care assessments for older adults at risk of falls. Such assessments should not rely exclusively on whether a person lives alone or has a small social network. Instead, they should include subjective loneliness, pain, perceived sleep quality, mobility limitations, frequency of outings, depressive symptoms and perceived relational support. This approach is consistent with a sociosanitary model of ageing in which clinical vulnerability and social vulnerability are understood as interdependent rather than separate domains.

### Limitations

Several limitations should be acknowledged. First, the cross-sectional design prevents the establishment of temporal or causal relationships between loneliness, sleep quality, pain and other clinical variables. Reverse causality is plausible, particularly in the associations between loneliness, sleep quality and pain. Loneliness may impair sleep and intensify pain perception, while poor sleep and severe pain may also reduce mobility, social participation and perceived relational availability.

Second, the study sample consisted of community-dwelling older adults at risk of falls and with a high burden of clinical vulnerability. Consequently, the prevalence estimate should not be generalised to all older adults in Andalusia or Spain. The findings are more appropriately interpreted as describing a clinically vulnerable subgroup in which fall-related vulnerability, pain, sleep problems and reduced social participation may converge.

Third, sleep quality was assessed using a self-reported item rather than a specific validated sleep questionnaire or objective sleep measure. Therefore, this variable reflects perceived sleep quality and may be affected by recall or reporting bias. Similarly, pain was assessed through self-report using the Visual Analogue Scale, which captures perceived pain intensity but does not provide information on pain duration, aetiology or clinical diagnosis.

Fourth, severe loneliness was defined using an operational threshold on the corrected UCLA-10 loneliness burden score. Although this approach facilitated the identification of participants with the highest loneliness burden, dichotomising a continuous psychosocial construct may reduce variability and should not be interpreted as equivalent to a clinical diagnosis.

Fifth, although the scoring direction of the UCLA-10 was reviewed and corrected during manuscript revision, this process highlights the importance of explicitly reporting item coding, total score range and cut-off criteria when using shortened loneliness instruments. All prevalence estimates, tables and interpretative statements were revised to ensure consistency between the corrected scoring direction, the operational definition of severe loneliness and the reported results.

Finally, the statistical model included a limited number of predictors to preserve model parsimony and stability. Other potentially relevant factors, such as intensity of social support, bereavement, duration of pain, fear of falling, cognitive function and use of community resources, were not fully explored and should be considered in future longitudinal research.

## 5. Conclusions

Severe loneliness was identified in 21.5% of community-dwelling older adults at risk of falls in Andalusia, using a corrected UCLA-10 loneliness burden score ranging from 0 to 30. This estimate indicates that severe loneliness is a clinically relevant condition in this vulnerable subgroup, although it should not be interpreted as representative of the broader older adult population in Andalusia.

In the adjusted model, poorer sleep quality and severe pain were the main independent clinical correlates of severe loneliness. Age and female sex were not statistically significant after adjustment for sleep quality and severe pain. These findings suggest that severe loneliness in this population is closely linked to modifiable clinical and functional vulnerability, particularly sleep disturbance and pain burden.

From a public health and primary care perspective, the results support the inclusion of loneliness screening within multidimensional assessments of frail older adults at risk of falls. Sleep problems and severe pain should be considered potential markers of social and emotional vulnerability rather than isolated clinical complaints. However, due to the cross-sectional design, the findings should not be interpreted causally. Longitudinal and interventional studies are needed to clarify temporal relationships and to determine whether integrated interventions addressing sleep, pain, mobility and social participation can contribute to reducing loneliness in later life.

## Figures and Tables

**Table 1:** Sociodemographic and clinical characteristics of participants according to severe loneliness status (N = 237).

Variable	Non-Severe Loneliness (*n* = 186)	Severe Loneliness (*n* = 51)	*p*-Value	Test
Age, years	78.94 ± 8.20	80.84 ± 8.84	0.130	Mann–Whitney U
Female sex	120 (64.5)	43 (84.3)	0.006	Fisher’s exact
Living alone	70 (37.6)	33 (64.7)	<0.001	Fisher’s exact
Low economic level	37 (19.9)	25 (49.0)	<0.001	Fisher’s exact
Good family relationship	181 (97.3)	43 (84.3)	0.002	Fisher’s exact
Sleep quality score	1.41 ± 1.35	0.45 ± 0.78	<0.001	Mann–Whitney U
Severe pain	44 (23.7)	31 (60.8)	<0.001	Fisher’s exact
History of depression	31 (16.7)	16 (31.4)	0.028	Fisher’s exact
Physical exercise score	0.75 ± 1.14	0.27 ± 0.75	0.005	Mann–Whitney U
Home outings score	1.47 ± 1.19	0.88 ± 1.05	0.002	Mann–Whitney U
Falls in previous year	82 (44.1)	30 (58.8)	0.081	Fisher’s exact
Polymedication	147 (79.0)	42 (82.4)	0.697	Fisher’s exact
Analgesic use	146 (78.5)	42 (82.4)	0.697	Fisher’s exact

Note: Values are presented as mean ± standard deviation or *n* (%), as appropriate. Severe loneliness was defined as a corrected UCLA-10 loneliness burden score ≥ 21.

**Table 2:** Highest item-level loneliness burden scores in the corrected UCLA-10 Loneliness Scale.

Loneliness Dimension/Item	Mean Score	Standard Deviation	Rank
Tolerance of solitude	2.10	0.91	1
Waiting for calls or letters	1.94	0.89	2
Inability to communicate	1.88	0.96	3
Desire for company	1.86	0.94	4
Unhappiness doing things alone	1.77	0.89	5

Note: Item scores are presented according to the corrected UCLA-10 scoring direction, where higher scores indicate greater loneliness burden. The table reports the items with the highest average loneliness burden in the study population.

**Table 3:** Multivariable logistic regression model for cross-sectional correlates of severe loneliness.

Predictor	Adjusted OR	95% CI	*p*-Value
Age (per incremental year)	1.03	0.99–1.07	0.184
Female sex (vs. male)	2.00	0.83–4.81	0.124
Sleep quality (per point improvement)	0.46	0.30–0.72	0.001
Severe pain	3.15	1.56–6.35	0.001

Note: Severe loneliness was defined as a corrected UCLA-10 loneliness burden score ≥ 21. OR = odds ratio; CI = confidence interval. Regression coefficients should be interpreted as cross-sectional associations.

## Data Availability

The data supporting the findings of this study are not publicly available because they concern older adults and may be subject to privacy and ethical restrictions. An anonymised dataset and analysis code may be made available from the corresponding author upon reasonable request and subject to approval by the relevant ethics committee.

## Abbreviations

The following abbreviations are used in this manuscript:

AUC	area under the receiver operating characteristic curve
CI	confidence interval
OR	odds ratio
STROBE	Strengthening the Reporting of Observational Studies in Epidemiology.
